# Supplementary material for: Antimicrobial mitochondrial reactive oxygen species induction by lung epithelial immunometabolic modulation
Source: PLoS Pathog. 2023 Sep 11;19(9):e1011138. doi: 10.1371/journal.ppat.1011138 (PMC10522048; doi:10.1371/journal.ppat.1011138)
Supplement: S2 Table — (DOCX) [file ppat.1011138.s002.docx]

**S2 Table. Regent sources and identifiers.**

| **REAGENT** | **SOURCE** | **IDENTIFIER** | |  |
| --- | --- | --- | --- | --- |
| **Antibodies** | | |  |  |
| AMPKα | Cell Signaling Technology | Cat# 2532; RRID: AB_330331 | | |
| ANT1/SLC25A4 | OriGene | Cat# TA321287 | | |
| ATP5A (51) | Santa Cruz Biotech | Cat# sc-136178; RRID: AB_2061764 | | |
| β-Actin (13E5) | Cell Signaling Technology | Cat# 4970; RRID: AB_2223172 | | |
| CAMP/Cramp | Novus Biologicals | Cat# NB100-98689; RRID: AB_1290695 | | |
| Citrate Synthase (D7V8B) | Cell Signaling Technology | Cat# 14309; RRID: AB_2665545 | | |
| COX IV (3E11) | Cell Signaling Technology | Cat# 4850; RRID: AB_2085424 | | |
| CPT1A | Abcam | Cat# ab220789 | | |
| ETFDH (D-2) | Santa Cruz Biotech | Cat# sc-515202 | | |
| GAPDH (14C10) | Cell Signaling Technology | Cat# 2118; RRID: AB_561053 | | |
| GPD2 | Abcam | Cat# ab188585 | | |
| Phospho-AMPK α-1,2 (Thr172, Thr183) | Invitrogen | Cat# PA5-36885; RRID: AB_2553800 | | |
| Phospho-AMPK α (Thr172) | Cell Signaling Technology | Cat# 50081; RRID: AB_2799368 | | |
| phospho-AMPKα1 (Ser485) | Cell Signaling Technology | Cat# 4184; RRID: AB_390759 | | |
| phospho-AMPKα2 | Abcam | Cat# ab129081; RRID: AB_11155357 | | |
| Phospho-ACC (Ser79) | Cell Signaling Technology | Cat# 3661; RRID: AB_330337 | | |
| SDHA (D6J9M) | Cell Signaling Technology | Cat# 11998; RRID: AB_2750900 | | |
| VDAC1 (B-6) | Santa Cruz Biotech | Cat# sc-390996; RRID: AB_2750920 | | |
| VDAC1 / Porin | Abcam | Cat# ab154856; RRID: AB_2687466 | | |
| CCSP | Millipore Sigma | Cat# ABS1673 | | |
| CD45-PE/Cy7 | BioLegend | Cat# 103114 | | |
| ICAM2-A647 | Invitrogen | Cat# A15452 | | |
| E-Cadherin-A488 | eBioscience | Cat# 53-3249-80 | | |
| **Chemicals** | | | | |
| **Pam2CSK4 and ODNs** |  |  | | |
| Pam2CSK4 | Invivogen | tlrl-pm2s-1 | | |
| ODN M362 | Invivogen | tlrl-m362 | | |
| ODN 2006-G5 | Invivogen | tlrl-2006g5 | | |
| ODN 2006 | Invivogen | tlrl-2006 | | |
| ODN 1826 | Invivogen | tlrl-1826 | | |
| ODN 2216 | Invivogen | tlrl-2216 | | |
| ODN 2395 | Invivogen | tlrl-2395 | | |
| ODN 1585 | Invivogen | tlrl-1585 | | |
| ODN D-SL01 | Invivogen | tlrl-dsl01 | | |
| ODN D-SL03 | Invivogen | tlrl-dsl03 | | |
| ODN M362 FITC | Invivogen | tlrl-m362f | | |
| FITC-labeled ODN 2395 | Invivogen | tlrl-2395f | | |
| FITC-labeled ODN 1826 | Invivogen | tlrl-1826f | | |
| Poly(dG:dC) | Invivogen | tlrl-pgcn | | |
| Poly(dA:dT) | Invivogen | tlrl-patn | | |
| ISD | Invivogen | tlrl-isdn | | |
| 5'ppp-dsRNA | Invivogen | tlrl-3prna | | |
| Poly(I:C) LMW | Invivogen | tlrl-picw | | |
| ssPolyU/ssRNA | Invivogen | tlrl-sspu | | |
| **Fluorescence Dyes and Luminescence Regents** | | | | |
| MitoProbe™ JC-1 | Thermo Fisher Scientific | M34152 | | |
| MitoSOX™ Red | Thermo Fisher Scientific | M36008 | | |
| MitoTracker® Red CMXRos | Thermo Fisher Scientific | M7512 | | |
| MitoProbe™ TMRM | Thermo Fisher Scientific | M20036 | | |
| CellROX™ Orange | Thermo Fisher Scientific | C10493 | | |
| ROSstar 550 | LI-COR | 926-20000 | | |
| MitoNeoD | AOBIOUS INC | AOB37866 | | |
| MCLA | Santa Cruz Biotech | sc-210442 | | |
| SYTOX Blue | Invitrogen | S34857 | | |
| **ROS Scavengers** |  |  | | |
| Mitoquinol/MitoQ | Cayman Chemical | 89950 | | |
| MitoTEMPO | Sigma | SML0737-5MG | | |
| **Mitochondria Inhibitors** |  |  | | |
| Rotenone | Cayman Chemical | 13995 | | |
| 2-Thenoyltrifluoroacetone | Sigma | T27006-25G | | |
| Atpenin A5 | Cayman Chemical | 11898 | | |
| Antimycin A | Sigma | A8674-25MG | | |
| Myxothiazol | Sigma | T5580-1MG | | |
| Stigmatellin | Sigma | 85865 | | |
| Sodium azide | Sigma | S2002-25G | | |
| Oligomycin A | Cayman Chemical | 11342 | | |
| FCCP | Cayman Chemical | 15218 | | |
| Dimethyl malonate | Santa Cruz Biotech | sc-239778 | | |
| α-Ketoglutaric acid | Sigma | 75890-25G | | |
| Oxaloacetic acid | Sigma | O4126-1G | | |
| Diphenyleneiodonium | Sigma | D2926-10MG | | |
| **Other Chemicals** |  |  | | |
| Carboxyatractyloside | Cayman Chemical | 21120 | | |
| Cyclosporin A | Cayman Chemical | 12088 | | |
| Erastin | Cayman Chemical | 17754 | | |
| VBIT-4 | Selleck Chemicals | S3544 | | |
| Etomoxir sodium salt hydrate | Sigma | E1905-5MG | | |
| **Enzymes and Kits** | | | | |
| Collagenase Type I | Worthington | LS004197 | | |
| Elastase | Worthington | LS002294 | | |
| DNase I | Worthington | LS002007 | | |
| CellTiter-Glo® 2.0 Assay | Promega | G9241 | | |
| AMP-Glo™ Assay | Promega | V5011 | | |
| NAD/NADH-Glo™ Assay | Promega | G9071 | | |
| NADP/NADPH-Glo™ Assay | Promega | G9081 | | |
| GSH/GSSG-Glo™ Assay | Promega | V6611 | | |
| PicoProbe™ ADP Assay Kit | BioVision | K211 | | |
| Mitochondrial Complex III Activity Assay | BioVision | K520 | | |
| Bioassay Systems QUANTICHROM ATPASE ASSAY | Thermo Fisher Scientific | 50-489-201 | | |
| Fatty Acid Oxidation (FAO) Assay | Biomedical Research Service | E-141 | | |
