# Supplementary material for: Antimicrobial mitochondrial reactive oxygen species induction by lung epithelial immunometabolic modulation
Source: PLoS Pathog. 2023 Sep 11;19(9):e1011138. doi: 10.1371/journal.ppat.1011138 (PMC10522048; doi:10.1371/journal.ppat.1011138)
Supplement: S3 Table — (DOCX) [file ppat.1011138.s003.docx]

**S3 Table. Experimental models.**

| **MODEL** | **SOURCE** | **IDENTIFIER** |  |
| --- | --- | --- | --- |
| **Cell Cultures** | | | |
| Primary Human Type II Alveolar Epithelial Cells | Accegen | ABC-TC5515 | |
| Primary Bronchial/Tracheal Epithelial Cells; Normal, Human | ATCC | PCS-300-010 | |
| HBEC3-KT cell line | John Minna | RRID:CVCL_X491 | |
| MLE-15 cell line | Jeffrey Whitsett | RRID:CVCL_D581 | |
| **Pathogens/Strains** | | | |
| *Pseudomonas aeruginosa* *PA103* | ATCC | 29260 | |
| **Mice/Strains** | | | |
| C57BL/6J (*Mus musculus*) | The Jackson Laboratory | Cat# JAX:000664; RRID:IMSR_JAX:000664 | |
| TLR9**^–/–^** (*Mus musculus*) | Shizuo Akira |  | |
| Prkaa1^fl^ (*Mus musculus*) | The Jackson Laboratory | Cat# JAX:014141; RRID:IMSR_JAX:014141 | |
| Prkaa2^fl^ (*Mus musculus*) | The Jackson Laboratory | Cat# JAX:014142; RRID:IMSR_JAX:014142 | |
| CMV mt–roGFP (*Mus musculus*) | D James Surmeier |  | |
| Sftpc-Cre (*Mus musculus*) | Brigid Hogan |  |  |
