# Supplementary material for: Antimicrobial mitochondrial reactive oxygen species induction by lung epithelial immunometabolic modulation
Source: PLoS Pathog. 2023 Sep 11;19(9):e1011138. doi: 10.1371/journal.ppat.1011138 (PMC10522048; doi:10.1371/journal.ppat.1011138)
Supplement: Attachment — Submitted filename: PLoS Pathogens Response to Reviewers 6-30-23.docx [file ppat.1011138.s021.docx]

**Responses to Editor’s Queries**

**Editor Query 1.** The first critical issue is your extrapolation to the whole lung. Although, your manuscript represents a large body of work showing that ODN can increase mtROS and thereby being part of the antimicrobial oxidative response of the human airway epithelium, I agree with the reviewer #1 that concerned only bronchial cells and moreover non-differentiated. For these reason, please pay particular attention to the comments #3 and #4 from the reviewer #1.

**Editor Response 1.**

Thank you for providing the opportunity to provide clarifying remarks regarding the cellular contributions to ODN-induced mtROS-dependent resistance. As advised by the Editor, this is extensively addressed in our responses to Reviewer 1’s Queries 1, 3 and 4 (and Reviewer 3’s Query 2). In short, we are comfortable extrapolating our findings to responses throughout the lungs. As described below, this confidence derives partly from data from two forthcoming papers. Since those data are not available for inclusion in this paper, we have added a total of 11 new experiments to demonstrate that ODN-induced mtROS and ODN-induced pathogen killing are observed in an exceptionally broad range of lung epithelial cells, including human and mouse cells, primary and immortalized cells, cells of airway and alveolar origin, and cells from differentiated and undifferentiated cultures in varying plating formats.

**EdQ2.** The second critical issue is the demonstrated link between mtROS production and the alteration of the electron chain transport.
Lines 90-92 (results from figure 1H): inhibiting complex V by oligomycin gives OCR due to proton leak since the ATP-based OCR is inhibited. Therefore, you showed increased OCR with ODN while inhibition by oligomycin. However, would it be possible to measure mtROS production in ODN+oligomycin and (ODN+antimycin or ODN+PBS). It will clearly show increased oxygen consumption by mtROS production.

**EdR2.** We thank the editor for this excellent suggestion. We have executed the proposed study. New Figure 1I shows induction of mtROS in the oligomycin + ODN treated cells, but not in the oligomycin + PBS or oligomycin + antimycin cells, exactly as predicted by the editor to support that the increased OCR reflects increased mtROS production.

**EdQ3.** Please address the reviewer #3’s major concerns #2 to confirm how you ruled out any ROS detection other than mtROS that could lead to falsely conclude to mtROS increase.

**EdR3.** As more extensively described in the response to Reviewer 3, we both reference our prior publications wherein we have used a total of 14 detectors of partially-overlapping ROS spectra to deduce the inducible ROS composition, and we have added new data to Figure S1 with an additional, recently reported detector (MitoNeoD) with high mtROS specificity. Although previously stated in the Introduction, we have further clarified that we have published that both mtROS and non-mtROS are required for protection in the Discussion. Thus, while we are confident that ODN-inducible mtROS are required for protection, we do not suggest lack of induction of non-mtROS. On the contrary, non-mtROS are most certainly induced.

**EdQ4.** Lines 168-169, you mentioned that AMPK is activated in response to ATP consumption. Thus, when ODN inhibits the ECT and subsequently the ATP production (results 2 and 3), AMPK is activated. Could you clarify why deletions of AMPK genes decreased ODN-induced mtROS?

**EdR4.** Thank you for the opportunity to clarify these findings. As demonstrated in revised Figures 3, 4 and 5, ODN treatment impedes VDAC1-dependent mitochondrial ATP/ADP antiport. This does not reduce ATP production per se, but it does reduce cytosolic ATP levels. Reduced cytosolic ATP promotes AMPK phosphorylation, resulting in activation of ACC, which activates CPT1 to promote mitochondrial fatty acid β-oxidation. Enhanced fatty acid β-oxidation increases acetyl-CoA-mediated input to the TCA cycle, with increased electron delivery to ETC complex II. We show that disruption of any the steps in this pathway (including deletion of AMPK genes) results in impaired mtROS production. In new Figure 5D we have added new data to explicitly show that AMPK gene deletion impairs fatty acid oxidation, just as we had already shown that AMPK gene deletion impaired mtROS induction and that inhibition of fatty acid oxidation impaired mtROS generation and ETC complex II activity. In addition to providing additional data, we have revised the Results text for clarity.

**EdQ5.** Line 377, please change “Luria-Bertani medium” for “Lysogeny broth medium” as G. Bertani himself explained that LB has been misconstrued to stand for his name (PMID: 14729683)

**EdR5.** Thank you for this correction. The text has been revised.

**EdQ6.** For the in vivo infection model (lines 370-375) please specify the time of nebulization that is required to ensure a full Pam or AND treatment as well as an effective PA inoculation.

**EdR6.**The inhaled treatments (PBS, single ligand Pam2, single ligand ODN, or combined Pam2 + ODN) are made as 10 ml solutions and nebulized for 40 min. Pseudomonas challenges are also reconstituted in 10 ml PBS, but nebulization is for 20 min. These exposure times were empirically-derived over the past 15+ years, optimized for reproducibility. We have revised the Methods for clarity.

**Reviewer 1 Summary.**

The report from Yongxing Wang et al. addresses the molecular mechanisms responsible for mitochondrial reactive oxygen species (mtROS) generation after epithelial challenge with TLR ligands. It continues the previous work of the team that developed antimicrobial agents in the context of respiratory research (pneumonia). The investigations are highly significant in the context of antimicrobial strategies for respiratory diseases. The background, the experimental design, and the general execution are robust. The intracellular metabolic cascade leading to ROS production is extremely well characterized by top-notch molecular approaches. The potential involvement of mitochondrial complexes II and III is convincingly described. The figures are appropriate and the results are accurately reported. The methods contained sufficient experimental details. I have only a few remarks that could improve clarity and complement the investigations.

**Reviewer 1 Major Issues:** (No Response)

**Reviewer 1 Minor Issues:**

**Reviewer 1 Query 1.** The title is somehow misleading for 2 reasons: (i) the work is mainly focused on HBEC3-KT cells that are originating from bronchi, therefore the extrapolation to the entire lung should be tackled with caution; (ii) the level of evidence dealing with the induction of mtROS by the modulators on the short kinetic that is presented hardly demonstrate “metabolic reprogramming”.

**Reviewer 1 Response 1.**

(i) Thank you for the opportunity to address this issue. The reviewer’s comments are understandable as much of the presented data are derived from HBEC3-KT cells that are, indeed, of bronchial origin. While the MLE-15 cells possess more alveolar characteristics than do HBEC3-KT cells, this alone is not convincing of extra-bronchial effects. We will begin by stating that we have additional data that indicate broad lung involvement that we cannot share here, as they are key data destined for inclusion in two other manuscripts. First, we observe both bronchial and alveolar ROS signal (L-012 luminescence) in lungs of mice exposed to Pam2ODN by nebulization. Second, when using cell-specific Cre to selectively disrupt signaling in (A) airway ciliated cells, (B) airway secretory cells, (C) type 1 alveolar cells or (D) type 2 alveolar cells, targeting any of the four cells partially impairs pathogen killing and host survival, indicating a critical role for both alveolar and airway cells in maximal inducible resistance. Since those data are not available for inclusion, we have added a total of 11 new experiments to show ODN-induced mtROS generation and pathogen killing by lung epithelial cells of human and mouse origin, both primary and immortalized, both airway and alveolar, both submerged and ALI, both differentiated and undifferentiated, both plated on collagen-coated wells and directly on plastic (revised Figure 1, new Figure S2, new Figure 7). Having modeled this response so broadly and reproducibly, we are comfortable describing the effect as occurring diffusely in lung epithelium.
(ii) Although we have previously described certain Pam2ODN-inducible effects that are exerted over the course of weeks to months, the Reviewer is correct that the currently presented studies are of much shorter duration. In view of this fair comment, we have changed the title and related content to describe “modulation” rather than “reprogramming.”

**R1Q2.** The production of ROS/mtROS is demonstrated but what about the release of these ROS? In addition, is there a way to test if those ROS are beneficial for the epithelial cells and non-toxic?

**R1R2.** These are not only excellent questions, they were central foci of prior work we have published. In particular, we previously showed in Kirkpatrick, mBio 2018 that Pam2ODN-treated (i.e., ROS-induced) lung epithelial cells survive better in the setting of infection than do untreated cells and that cell survival of uninfected cells is not harmed by Pam2ODN treatment (Kirkpatrick Figures 3 and 4). In the same paper, we show that the ROS are released from the cells by means of cell-impermeant detectors (Kirkpatrick Figures 5 and 7). We have also shown in many prior papers that Pam2ODN treatment improves mouse survival of a wide range of infectious challenges without demonstrating untoward effects on the animals. We have revised the Discussion text to better reflect these elements. We also previously noted in the Discussion that nebulization of this treatment is non-toxic in human subjects.

**R1Q3.** Some more background and details regarding the origin of the epithelial cells and the related hypothesis that is tested should be given in the text. For example, HBEC3 are mainly used, since they are originating from bronchi they may be ideally suited to analyze the first intra-pulmonary contact of the bacteria but pneumonia particularly affects the alveoli (only MLE-15 was used for only one experimental approach). Is it possible to reproduce the most important findings in another cellular model that would represent small airways or alveoli? It should be at least discussed in the manuscript.

**R1R3.**

As noted in response to the Reviewer’s Query 1, we have added 11 new experiments to demonstrate that airway and alveolar cells respond similarly to ODN to generate pathogen-killing ROS (including both mouse and human alveolar cells, suggested here). Thus, we are confident that pathogen killing is inducible at both airway and alveolar levels. However, the reviewer raises the interesting (and likely correct) notion that pathogen killing on first interaction in the airway may have different net effects than pathogen killing at the alveolar level. Accordingly, we have also added further treatment of this topic to the Results.

**R1Q4.** The authors should also discuss the limitations of their work regarding the in vitro model that was chosen. HBEC3 are non-differentiated cells, whereas the bacteria first encounter differentiated cells in the airways. In addition, it is not clear why and when cell culture was performed on collagen-coated tissue culture plates and transwells since no data that is presented here seem to discuss influencing parameters such as proliferative state of the cells, level of differentiation, or even general morphology/phenotype. If transwells were used, did the authors try to distinguish non-differentiated vs differentiated airway epithelial cells to test the differential ROS production?

**R1R4.** These are also all excellent observations. As described in our responses to Query 1 and Query 3, we have tested all of the combinations suggested by the reviewer and find no conditions where we cannot induce both mtROS production and pathogen killing. We provide all requested data in revised Figure 1, new Figure S2, and new Figure 7. As requested, evidence of differentiation of our transwell cultures is also included in new Figure S2. Further, we have added additional coverage of model selection and its implications to the Results.

**R1Q5.** The full datasets with all the proteins should be provided in supplemental data regarding Figure 3D and Figure 4A.

**R1R5.** As requested, the full LC-MS dataset for Figure 3D has been added as new Supplemental Table 1. As in the Data Availability Statement, the RPPA dataset for Figure 4A is publicly available at [www.github.com/evanslaboratory/Datasource](http://www.github.com/evanslaboratory/Datasource). Additionally, related content has been recently published in Pantaleon Garcia, NAR Genom Bioinform 2022 (AMPK is not addressed in that paper). The appropriate citation has been added.

**R1Q6.** The manuscript is generally very well written. Nonetheless, there are a few typos to correct.

**R1R6.** We have carefully reviewed the document and made corrections.

**R1Q7.** Is there a label that is not correct in Figure S1A or S1B? As it is I do not understand the difference between those 2 panels.

**R1R7.** To avoid confusion, we have removed the previous Figure S1B. (New Figure S1B is a different detector and that change is unrelated to this query.)

**R1Q8.** The scale bars on the IF do not appear correct or the cells that are shown would reach a few hundred µm.

**R1R8.** Thank you for catching this. The legend has been properly updated.

**R1Q9.** Is it possible to apply a statistical method to analyze the curves in OCR measurements (F1H, F3L, F4J, F5C, FS12G) and F5D-G?

**R1R9.** This is certainly possible. We have provided the appropriate statistical comparisons for all groups in each OCR curve (Figure 1H, Figure 3L, Figure 5F, Figure 6D, and Figure S14G).

**R1Q10.** Is it possible to show close inserts for F4D? In addition, it would be interesting to explore the alveoli.

**R1R10.** Inserts have been added to the three panels in Figure 4D, allowing closer inspection of the airways. Phospho-AMPK fluorescence is also evident in the alveolar space, though some of this is obscured by the new inserts. We now specifically mention in the Results that staining in both regions is observed.

**Reviewer 2 Summary.**

Evans and colleagues have developed and functionally characterized a combination of two innate immune stimulators (ODN M362 and Pam2) that induces epithelial resistance against a broad range of opportunistic bacterial and fungal pathogens. In prior work, the authors showed that the induction of mitohondrial ROS is critical to the efficacy of this immunomodulatory strategy that is being tested in human clinical trials. This new manuscript describes the molecular links that bridge Pam2/ODN M362 and mtROS, with the surprising finding that ODN M362 directly binds to the mitochondrial voltage-dependent anion-sensitive channel (VDAC1) in a manner that is independent of TLR9 signaling. This interaction results in metabolic reprograming in mitochondria, and alters cellular nucleotide distribution which in turn activates the AMPK-ACC and fatty acid oxidation. These events promote electron flux through the ETC and the generation of mtROS; ODN M362-dependent disruption of these events results in impaired epithelial mtROS generation and loss of in vivo activity of the ODN M362/Pam 2 activity, resulting in impaired lung host defenses against Pseudomonas challenge in experimental models of disease. Overall, this is an elegant and well-developed study that is relevant for understanding epithelial regulation of innate immunity (and its therapeutic manipulation).

**Reviewer 2 Major Issues:** I do not recommend additional experimentation for acceptance of this manuscript.

**Reviewer 2 Minor Issues:**

**Reviewer 2 Query 1.** Figure 2B - please quantify Western blot bands.

**Reviewer 2 Response 1.** This is a great suggestion. Densitometry data for all Western blots and pulldowns have been added to revised Figures S7 and S10.

**R2Q2.** Line 137-140. Re pulldown assays: Is VDAC1 one of the bands in the 35-50 kDa range - Fig. 3D? Please quantify increase in IP of VDAC1 or ANT1 as a function of ODN exposure.

**R2R2.** Yes! VDAC1 is one of the peptides pulled down in those bands, though it does not necessarily account for an entire band. We have revised the Results text to clarify this point. As above, densitometry data for all Western blots and pulldowns have been added to revised Figures S7 and S10. Also, in response to a query from Reviewer 1, new Table S1 includes all peptides that were differentially pulled down in Figure 3D.

**R2Q3.** See point 2 for Fig. 4B - please quantify.

**R2R3.** As above, densitometry data for all Western blots and pulldowns have been added to revised Figures S7 and S10.

**R2Q4.** Figure 4 contains an enormous amount of data and recommend splitting in two figures (panels A - F in one figure; panels G - P in second figure; panel P could be the first panel in the second figure to aid the reader in absorbing the ensuing experiments - currently displayed in panels G - O).

**R2R4.** As suggested by the reviewer, former Figure 4 has been split into new Figures 4 and 5 and the summary panel (formerly Figure 4N) is now Figure 5A.

**R2Q5.** Similar considerations for current Fig. 5 - helpful to lead with model - panel N - and then associated data.

**R2R5.** As suggested by the reviewer, summary Figure 5N is now Figure 6A.

**Reviewer 3 Summary.**

This manuscript investigates how ODN induces mitochondrial ROS (mtROS) production via metabolic reprogramming, alters mitochondrial electron transport chain (ETC) activity in a mitochondrial membrane potential (ΔΨm)-dependent manner. They show that antimicrobial ROS are induced from lung epithelial cells by interactions of CpG oligodeoxynucleotides (ODNs) with mitochondrial voltage-dependent anion channel 1 (VDAC1) without dependence on Toll-like receptor 9 (TLR9). The ODN-VDAC1 interaction alters cellular ATP/ADP/AMP localization, increases delivery of electrons to the electron transport chain, enhances mitochondrial membrane potential, and differentially modulates ETC complex activities. These combined effects promote leak of electrons from ETC complex III, resulting in superoxide formation. They conclude that ODN-induced mitochondrial ROS may yield protective antibacterial effects. Although the studies are interesting and provide some understanding of protective effects of ODN on host immune response, the studies are not enough to conclude that ODN induced mitochondrial ROS generation in epithelial cells is the sole contributor to host protection in vivo in a model of lung infection with P. aeruginosa.

**Reviewer 3 Major Issues:**

**Reviewer 3 Query 1.** The figures that show the seahorse analysis demonstrate effects of oligomycin and antimycin. The entire graph of cellular respiration needs to be shown with basal, maximal OCR, spare respiratory capacity and ATP production. In addition these should be accompanied with graphs and quantification

**Reviewer 3 Response 1.**

The Reviewer accurately describes standard outputs of the Seahorse XF Mito Stress Kit using oligomycin, FCCP, antimycin and rotenone. However, that protocol is chiefly optimized for assessing oxidative phosphorylation in non-epithelial cells, whereas our interest was in detecting oxygen consumption by superoxide formation when oxidative phosphorylation is inhibited. As such, we performed the maneuvers required to detect our signal of interest, rather than the default protocol. However, as suggested by the Editor, we have demonstrated that the increased oxygen consumption correlates with the increased mtROS production in new Figure 1I.

**R3Q2.** The investigators use both tracheal and bronchial epithelial cells. The rationale for use of these cells needs to be included

**R3R2.** We thank the reviewer for this important and accurate comment. As noted above in responses to the Editor and Reviewer 1, we have provided copious new data showing that the ODN-induced responses can be generated from all tested lung epithelial cells. Further, in direct response to this comment, we have also provided further rationale for the models used in the Results section.

**R3Q3.** In Figure 2 F and G they show the expression of NADH and NADPH, however, none of the experiments include quantification of non-mitochondrial ROS. These should be done in combination with infection of bacteria in vitro.

**R3R3.** This is an important consideration that the Reviewer points out could be better explained in the manuscript. The authors completely agree about the essential role of non-mtROS in the response. That both mtROS and non-mtROS are required for inducible epithelial resistance is the central message of two of our recent papers (viral models: Kirkpatrick, mBio 2018; bacterial models: Ware, PLoS ONE 2019). We regard that point as settled and in the current work we focus on the mechanisms of mtROS induction, rather than re-proving that mtROS are required for protection. In explicit response to the reviewer’s comment, we have added the requested new data (Figure S16) showing the induction of non-mtROS in the presence/absence of bacteria. We have also provided another layer of specificity to our mtROS assessments by adding new data with MitoNeoD (new Figure S1B). Perhaps more importantly, although this was already stated in the Introduction (Lines 54-55), we have revised the Discussion to emphasize the Reviewer’s point that non-mtROS contribute to the overall protective response, although we focus on the mechanisms of mtROS induction in this work.

**R3Q4.** Most of the in vitro studies are performed with ODN alone, it will interesting to include experiments with P. aeruginosa and ODN

**R3R4.** This is, indeed, an interesting suggestion! We have added this experiment as new Figure S16C, showing that *P. aeruginosa* does not influence ODN-induced mtROS levels.

**R3Q5.** Many of the conclusions are based on use of VDAC1, ANT1, mPTP inhibitors, off target effects of these reagents should be considered

**R3R5.** The reviewer is absolutely correct that off-target effects must be considered whenever pharmacologically manipulating a biological system. Although our confidence in the presented findings is enhanced by the congruence of the findings using the noted VDAC1, ANT1 and mPTP inhibitors (i.e., we would not expect the same off-target effects for three pharmacologically-distinct agents that perturb the same system at three different points), we have expanded our acknowledgement of this limitation in the Results section. Further, we have added new data using another VDAC1 inhibitor, VBIT-4, that inhibits VDAC1 by different mechanisms than erastin or cyclosporin A yet yields parallel effects on mtROS production, mitochondrial membrane potential and pathogen killing (new Figure S9 and new Figure 7I). Although this does not formally eliminate the possibility of off-target effects as drivers of the observed responses (as acknowledged in the revised text) it makes that statistically much less likely as the full explanation.

**R3Q6.** The in vivo experiments in Figure 6 show a protective effect of Pam2-ODN on P. aeruginosa infection. From the data and design of experiments it is difficult to conclude that this would be solely due to mitochondrial ROS generation by ODN in epithelial cells in vivo. Multiple cell types particularly phagocytes such as neutrophils, macrophages contribute to host defense in bacterial infections. Although ODN was administered as nebulization, it is likely to have effects on other immune cells.

**R3R6.** The authors agree with every element of this comment. As noted in R3R3, we make no claim that only mtROS contribute to the protective response. Similarly, we fully acknowledge that other cells types can both respond to ODN and contribute to the vast array of protective host responses. Despite all these other potential contributing elements, we find it quite remarkable that we can still substantially impair protection by only disrupting mtROS generation (i.e, mtROS are required for protection). Further, by showing here that epithelial cells in isolation can be induced to kill pathogens in a mtROS-dependent manner, we again demonstrate epithelial sufficiency of this response. In response to this comment, we have clarified in the Discussion that protection does not solely depend upon epithelial mtROS. Balancing this acknowledgement, we also note that we have published that we can still generate profound in vivo pneumonia protection despite depleting the other potentially-responding cells referenced by the reviewer (Cleaver, Mucosal Immunology 2014, Leiva-Juarez, Blood 2016), thereby underscoring the key role of epithelial cells in inducible resistance.

**Reviewer 3 Minor Issues:** (No Response)
